# Novel Low-Cost Endoscopic Cap for Esophageal Foreign Objects: A Case Report

**DOI:** 10.1097/MD.0000000000000796

**Published:** 2015-05-01

**Authors:** King-Wah Chiu, Lung-Sheng Lu, Tsung-Chin Wu, Shue-Shian Chiou

**Affiliations:** From the Division of Gastroenterology and Hepatology, Department of Internal Medicine, Kaohsiung Chang Gung Memorial Hospital (K-WC, L-SL, T-CW, S-SC); and College of Medicine, Chang Gung University, Taiwan, Republic of China (K-WC).

## Abstract

A 57-year-old man presented to the hospital because of swallowing of a small marble precipitated by a hallucination. He subsequently developed chest discomfort. He had a history of psychiatric problem and an esophageal corrosive injury complicated by stricture of the middle esophagus.

This report describes the novel idea of endoscopic intervention for the retrieval of an esophageal foreign body. Its inventiveness and the use of limited resources, by adapting a 30-mm aseptic common tubing into an endoscopic retrieving device, make the method novel. This novel low-cost endoscopic cap (NLCEC) was adapted to 25 mm of the front end of the endoscope, with 5 mm maintained for the soft part to prevent esophageal mucosal injury during the retrieval process. An 8-mm green marble was found impacted in the esophagus 32 cm from the incisors. The use of forced suction allowed for the successful retrieval of the marble within minutes. The patient had an uneventful recovery without any serious complications.

This NLCEC may be a viable and safe tool for the endoscopic retrieval of esophageal foreign objects without general anesthesia. This innovative design is beneficial in terms of patient safety, easy preparation, and low cost.

## INTRODUCTION

A patient swallowing a foreign object (FO) is an emergency situation and an acute issue for endoscopists. Many endoscopic devices are recommended for the retrieval of an ingested foreign body; however, the narrow working space remains a considerable challenge. This case report describes a unique, cost-effective, and novel approach for the retrieval of an esophageal foreign body (a small marble) in an otherwise hemodynamically stable patient.

## PRESENTING CONCERNS

The patient was a 57-year-old Taiwanese man who was a general worker. He denied having major systemic diseases but had a major depressive disorder associated with occasional psychiatric comorbidity, for which he was treated with quetiapine (200 mg/day) and seen regularly at the outpatient clinic. Five years ago, he experienced a corrosive injury to the esophagus that was complicated by a focal stricture at the level of 32 cm of the esophagus from the incisors. He did not receive any intervention such as esophageal dilations. Since then, he had generally been on a diet of soft foods such as rice, meat, and fruits. He occasionally experienced vomiting when eating too fast or eating large amount of foods within a short period. He also had bouts of hallucination and insomnia, but with no evidence of an impaired quality of life or effects of suicidal ideation and attempts. On the morning of the incident, he heard a voice telling him to swallow a small marble. After he did so, he experienced chest pain, dysphagia, and nausea. He had no paresthesia or neck pain.

## CLINICAL FINDINGS

The patient was transferred by ambulance to the emergency room. He was conscious and coherent, and without respiratory distress. He easily described what he was experiencing. He had stable vital signs, including a blood pressure of 124/78 mm Hg, respiratory rate of 12 breaths/minute, pulse rate of 62 beats/minute, and temperature of 37°C. Physical examination produced unremarkable findings, with no wheezing on auscultation. Blood examination showed normal hemogram and biochemistry values, and chest radiography revealed no significant findings.

## DIAGNOSTIC FOCUS AND ASSESSMENT

**FIGURE 1:**
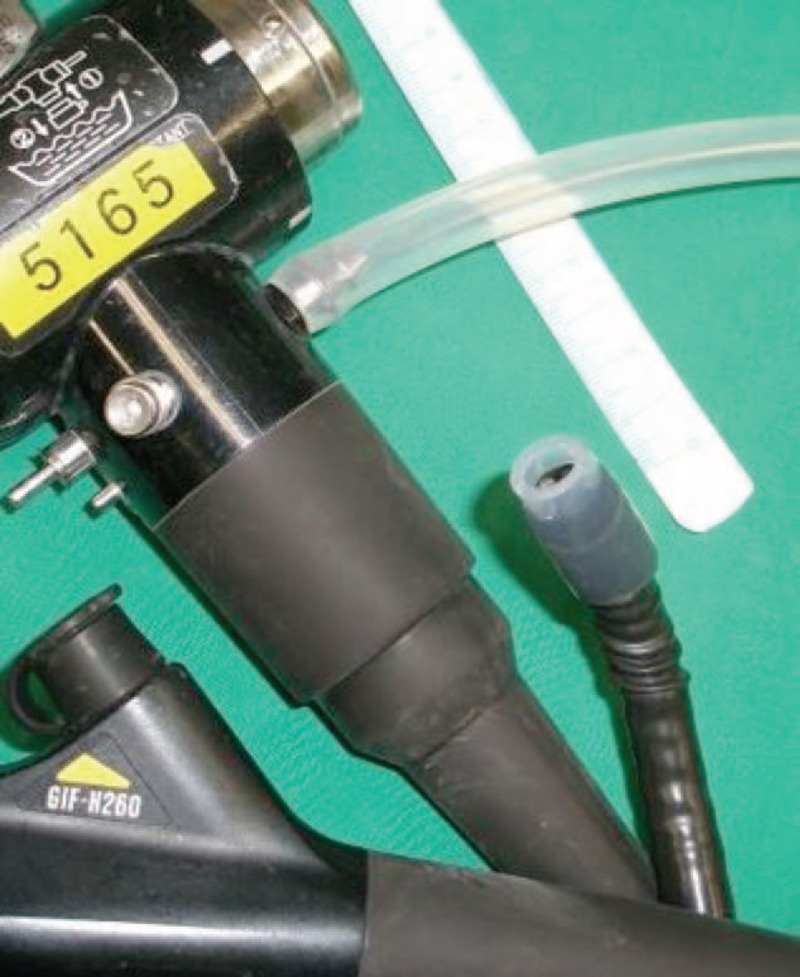
The novel low-cost endoscopic cap (NLCEC) is picked up by using a soft elastic suction tube that is used to connect the endoscope and suction bottle.

**FIGURE 2:**
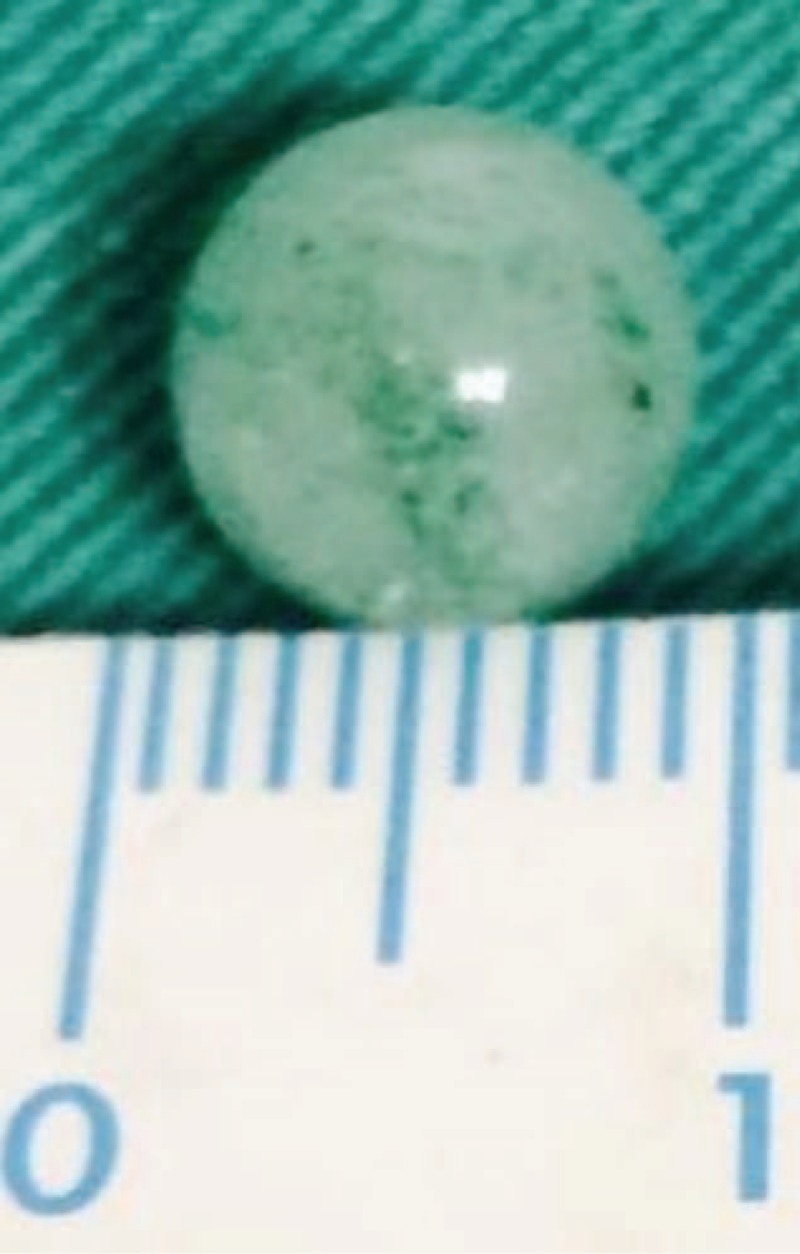
A greenish marble around 8 mm in diameter was retrieved.

Because of the patient's unique characteristics, general anesthesia was not recommended. Upper endoscopy under sedation was performed with an Olympus H-260 gastroscope. Before the endoscopic intervention, the limited resources were circumvented by utilizing a 30-mm short-length aseptic common tubing as a novel low-cost endoscopic cap (NLCEC). The NLCEC was adapted to 25 mm of the front end of the endoscope, and a 5 mm soft part was maintained to prevent esophageal mucosal damage during the retrieval process (Figure 1). A greenish marble (diameter, ∼8 mm) was found impacted at the stricture level of the esophagus 32 cm from the incisors. The use of forced suction allowed for the successful retrieval of the marble within minutes (Figure 2).

## FOLLOW-UP AND OUTCOMES

After the endoscopic retrieval of the esophageal foreign body, the patient was sent back to the emergency room for further observation. We observed him while he recovered from the endoscopy procedure. He had no adverse events such as bleeding, perforation, or infection. After an uneventful recovery within 4 hours of clinical observation, he was discharged from the emergency room.

## DISCUSSION

The American Society for Gastrointestinal Endoscopy produced guidelines in 2011 to aid decision making in retrieving FOs from the body.^1,2^ Various devices have been used for foreign body retrieval, including forceps, polypectomy snares, polyp graspers, and nets. In this present case, all of those devices cannot be used in a narrowing space like an esophagus with a stricture. The idea of an NLCEC is derived from the clinical use of banding ligation for bleeding esophageal varices. This technique is not performed frequently; however, some endoscopists have advocated its use for the retrieval of esophageal foreign bodies. It is a unique, cost-effective, easy-to-prepare, and novel approach for the successful retrieval of an esophageal FO of a certain size and type, in this case a small marble, in an otherwise hemodynamically stable patient.

Safety is important in performing invasive medical procedures. Instruments for esophageal banding ligation, such as the overtube and endoscope cap, were not used in this patient to avoid esophageal perforation or esophageal mucosal damage during the retrieval process. In contrast to the hardness of such devices, the NLCEC is soft and can protect the weak mucosa of the stricture, thereby avoiding mucosal injury. Because of its elastic characteristic, the diameter of the suction tube adapted to the front end of the endoscope does not require overtube guidance, which is an additional device used to prevent the FO from falling into the trachea. The complete tight suction supplements the close contact of the tube to the round surface of the hard object (Table 1). The suction power is strong enough to retrieve the hard marble through the pharynx, and the elastic cap can be adjusted to achieve a complete contact angle with the hard FO when the suction power is turned on. In contrast, the hard cap of the resection device or the esophageal banding ligation device is not recommended because the hard FO surface will not be in close contact with the suction cap without the correct angle.

**TABLE 1:**
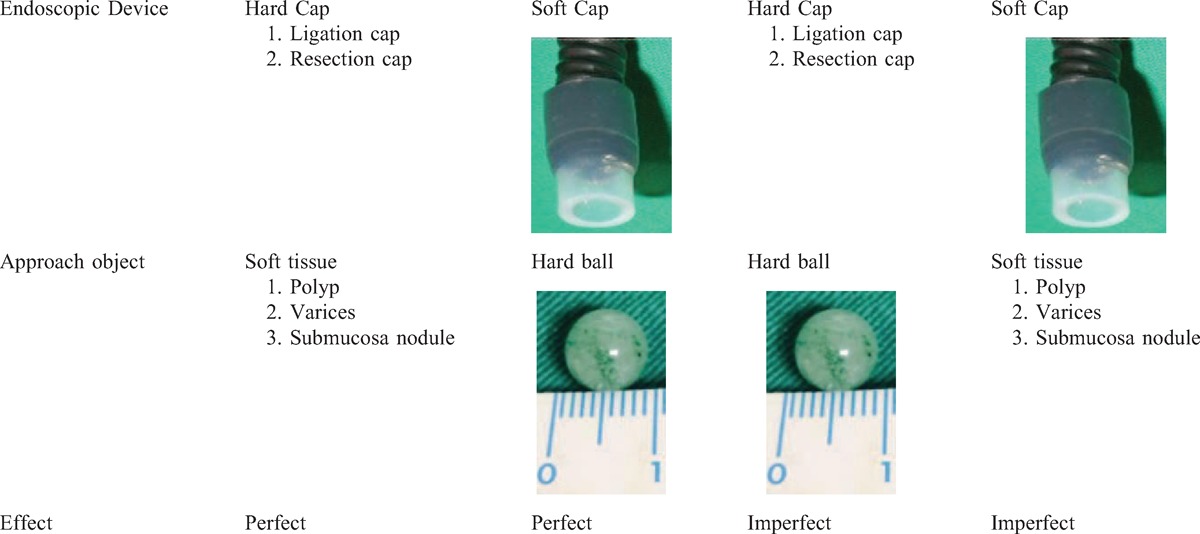
Mechanism of the Suitability of the Endoscopic Cap for Retrieving the Foreign Body

Lastly, the approach is easy and lasts within a couple of seconds. Thus, general anesthesia and intubation are not needed,^3^ avoiding the risks associated with anesthesia.^4^ The advantages of this method are important, especially because some medical procedures are not covered by government insurance. This NLCEC is cost-effective and is much cheaper (free of charge) than the conventional resection endoscopic cap (US $20–30). It can be one of the best choices for a medical service in a developing country.^5^ This innovative method should benefit both patients and endoscopists in clinical practice.

For the postprocedural management, a chest roentgenogram and esophagogram with a water-soluble contrast medium can help examine the stricture and confirm the successful retrieval of the esophageal FO.

However, the NLCEC as a retrieval device may be limited by the characteristics of the FO. If the surface of the FO is not smooth, the consistency is soft, or the size is too big, the FO should be removed with other endoscopic retrieval devices.

### Take-Away Messages

The first priority of the NLCEC is safety for the patient. The soft part of the common tubing is viable for the hard round surface of the FO and protects the surrounding esophageal mucosa during the retrieval process. The first consideration in using the NLCEC device for endoscopic retrieval is the small and/or narrowed space of the esophageal gastrointestinal tract. Because of the easy and fast preparation, conventional endoscopy without general anesthesia is much more beneficial for otherwise hemodynamically stable patients.
